# Hippocampal dysfunction in the pathophysiology of schizophrenia: a selective review and hypothesis for early detection and intervention

**DOI:** 10.1038/mp.2017.249

**Published:** 2018-01-09

**Authors:** JA Lieberman, RR Girgis, G Brucato, H Moore, F Provenzano, L Kegeles, D Javitt, J Kantrowitz, MM Wall, CM Corcoran, SA Schobel, SA Small

**Affiliations:** 1 Department of Psychiatry, College of Physicians and Surgeons, Columbia University, New York, NY, USA; 2 New York State Psychiatric Institute, New York, NY, USA; 3 Department of Neurology, College of Physicians and Surgeons, Columbia University, New York, NY, USA; 4 Department of Biostatistics, Mailman School of Public Health, Columbia University, New York, NY, USA; 5 Department of Radiology, College of Physicians & Surgeons, Columbia University, New York, NY, USA

## Abstract

Scientists have long sought to characterize the pathophysiologic basis of schizophrenia and develop biomarkers that could identify the illness. Extensive postmortem and *in vivo* neuroimaging research has described the early involvement of the hippocampus in the pathophysiology of schizophrenia. In this context, we have developed a hypothesis that describes the evolution of schizophrenia—from the premorbid through the prodromal stages to syndromal psychosis—and posits dysregulation of glutamate neurotransmission beginning in the CA1 region of the hippocampus as inducing attenuated psychotic symptoms and initiating the transition to syndromal psychosis. As the illness progresses, this pathological process expands to other regions of the hippocampal circuit and projection fields in other anatomic areas including the frontal cortex, and induces an atrophic process in which hippocampal neuropil is reduced and interneurons are lost. This paper will describe the studies of our group and other investigators supporting this pathophysiological hypothesis, as well as its implications for early detection and therapeutic intervention.

## INTRODUCTION

### Background and historical context

Scientists have been challenged to elucidate the pathological basis of mental illness. In the 19th century, when medicine embraced scientific methods and technology to identify the pathologic basis of human disease enabling clinical diagnosis to advance beyond descriptive phenomenology and syndromal definitions (e.g., dropsy, apoplexy, diabetes mellitus and insipidus), psychiatry lagged behind.

Identifying the pathological bases of disease led to the categorical distinctions of functional and organic conditions, with visualizing the pathology—typically in postmortem tissue—representing the cardinal criterion. Consequently, most neuropsychiatric disorders were considered “functional” as there were no apparent neuropathological stigmata. It was not until 1906 when a new class of dyes developed for industrial use enabled Alois Alzheimer, a psychiatrist trained in neuropathology working at the University of Munich under Emil Kraepelin, the founder of modern psychiatric diagnoses, to apply a special stain to post-mortem brain tissue, that the first pathological diagnosis of a mental disorder was established. The histologic signature of senile plaques and neurofibrillary tangles subsequently became the sine qua non of the eponymous neurodegenerative disease.

This scientific advance illustrated how progress in biomedical research depends on technological developments. However, technology, such as it was at the time, could not enable Kraepelin to identify the neuropathological basis of other “functional” brain disorders, and he was relegated to make his seminal distinction between dementia praecox and manic-depressive illness by painstaking description of symptoms and illness course. Since then, researchers have continued to search for the neuropathological features of mental disorders. While some pathologic features specific to mental disorders (e.g., schizophrenia) have been identified, these have not risen to the level of being diagnostic.^1–4^

Although, etiologic and pathophysiologic hypotheses of schizophrenia have subsequently been developed, they have neither yielded biologic measures that were diagnostic nor fully elucidated the pathophysiologic basis of the illness. In particular, the early phase of the illness, and how people evolve from ostensibly normal in their premorbid phase to meet DSM criteria for schizophrenia and related psychotic disorders, is incompletely characterized and poorly understood.

Extensive evidence indicates that schizophrenia is a complex neurodevelopmental disorder reflecting the interplay of genetics and the environment.^5,6^ That is to say that some combination of risk alleles (in the form of SNPs) and *de novo* or inherited mutations or copy number variants, affects brain development forming the diathesis for schizophrenia with contributory environmental factors. It is also highly likely that there may be multiple different etiologies that phenotypically converge to induce the symptoms of schizophrenia.^7^

While genetic and early developmental factors contribute to the diathesis of schizophrenia, its phenotype is not overtly manifest until after puberty, with its symptoms generally emerging gradually or iteratively from adolescence through young adulthood (Supplementary Figure S1).

Therapeutic pessimism historically colored perspectives on the prognoses and treatment of schizophrenia, but more recent studies (in the 1990s and early 21st century) of first episode and early stage of illness patients revealed that symptom remission and recovery are possible if patients receive prompt, appropriate treatment.^8,9^ This research also revealed a key feature of schizophrenia. Treatment response and outcome were significantly affected by the duration of active illness prior to treatment. Longer duration of psychotic symptoms prior to treatment intervention is associated to longer time to, and lower rates of, symptom remission.^10–13^ This suggested that the active psychotic phase of the illness reflects an ongoing pathophysiological process that can impair patients’ capacity for therapeutic response and good outcomes.^14^

This body of research led to efforts to develop innovative treatment strategies for patients in the early stages of psychotic disorders designed to reduce the duration of untreated illness and promote engagement in sustained treatment and reduce non-adherence and relapse.^15,16^ While current clinical methods are sufficiently robust for diagnosing and effectively treating first episode psychosis patients, thereby establishing a standard of care of this stage of illness, the methodology for subjects suspected of being in the antecedent “prodromal” stages of the schizophrenia—so-called clinical high-risk state—is inadequate for clinical implementation. Our methods for case identification are insufficiently specific, and we do not know what are the optimal treatments for people approaching or at the incipient stage of their illness.^17^ Previous studies have shown that at most, 30% of people who met criteria for being at clinical high risk to develop schizophrenia actually go on to develop syndromal psychosis.^18^ Thus, the case identification criteria, which represent the state of the art, have the potential for a 70% or higher false positive rate (people identified as clinical high risk but do not develop bona fide psychotic disorders). Therefore, we risk mislabeling and treating them, possibly unnecessarily, to alleviate their attenuated psychotic symptoms and prevent the onset of schizophrenia.

### A pathophysiologic hypothesis of schizophrenia onset

To advance our knowledge of psychotic disorders and improve the methodology for early detection and intervention in the presyndromal phase of schizophrenia, we developed a pathophysiologic model that characterizes the progression of schizophrenia from the premorbid through the prodromal stages to syndromal psychosis. This model posits dysregulation of glutamate neurotransmission occurring in the CA1 region of the hippocampus which elevates neuronal activity reflected in metabolism and blood flow, and in doing so elicits attenuated psychotic symptoms and initiates the prodromal stage of schizophrenia. As this persists, it drives the transition process to the later prodromal stage and subsequently syndromal psychosis. As the incipient illness progresses, this dysfunction expands to projection fields within and external to the hippocampus and frontal cortex, and causes an atrophic process in which the neuropil of hippocampal cells is reduced and interneurons are lost. This paper will describe and review the research that underpins this model, hippocampal pathophysiology in the onset of schizophrenia (Supplementary Figure S2). The evidence supporting this model is substantial, consistent and derives from numerous investigators.^3,6,9–30,36^ Our contributions to this body of research began with studies of the pathophysiological progression of schizophrenia^11–13,31^ and more recently on the pathogenic mechanisms of the onset of the illness.^19,20,36^

### Diagnostic biomarker

Our aim was to elucidate the pathophysiologic mechanism leading to the onset of schizophrenia and develop a pharmacologic intervention. We began by developing a biomarker to facilitate the diagnosis of people at clinical high risk for psychotic disorders. We focused on the hippocampus because previous neuroimaging research has demonstrated structural (volume reduction, shape anomalies) and functional abnormalities (increased metabolism measured by blood flow and glucose metabolism) in the medial temporal lobe structures of patients with schizophrenia (specifically the CA1 and subiculum subregions of the anterior hippocampus).^3,4,18–30^ The overlap between the anatomical pattern of hippocampal hypermetabolism and structural changes suggested that these abnormalities might stem from a common pathophysiologic mechanism. However, as these findings came from different studies with separate patient samples, their concordance within subjects was unknown. Moreover, the temporal sequence of these pathologic features and whether they are progressive was unclear.^32,33^

## MATERIALS AND METHODS

### Prospective study of clinical high-risk subjects

We longitudinally assessed 25 subjects who met criteria for ‘clinical high risk’ (CHR) for psychosis, ages 15–30. Subjects were ascertained using the Structured Interview for Psychosis Risk Symptoms SIPS^34^ and enrolled if they met criteria for at least one of the psychosis-risk syndromes, as defined by the SIPS^34^: (1) Attenuated positive symptom psychosis-risk syndrome (APSS); (2) Genetic risk and deterioration psychosis-risk syndrome (GRDS); and/or (3) Brief intermittent psychotic psychosis-risk syndrome (BIPS), and were followed for a minimum of 2 years or until they met criteria for a DSM-5 psychotic disorder.^19,35^

Nineteen control subjects were also recruited. Eligibility criteria for these subjects were the same as for the CHR subjects, with the exception that none scored higher than a 2 on any SIPS Positive symptom or met criteria for a past or current DSM Axis I disorder at baseline. All subjects were medically healthy. Approval from the NYSPI Institutional Review Board was obtained prior to initiating research. All adult subjects provided written informed consent and minors provided written assent, with written informed consent given by one or both parents.

In addition to clinical ratings of psychopathology (with the structured interview for psychosis risk syndromes (SIPS), subjects were assessed with MRI measures of structure and function using gadolinium (a paramagnetic contrast agent) to provide high-resolution maps of the spatial and temporal pattern of hippocampal metabolism and structure^19,35,36^ and followed for 2 years or conversion to a psychotic disorder at which time they were reassessed with the same measures as at study entry. Embedded within the SIPS is the scale of prodromal symptoms (SOPS) which is a 19-item scale organized around 5 domains positive symptoms (5 items, including unusual thought content/delusional Ideas), negative symptoms (6 items, including social anhedonia and avolition), disorganized symptoms (4 items, including odd behavior and bizarre thinking), and general symptoms (4 items, including sleep disturbance and dysphoric mood).

### Hippocampal function

**Figure 1:**
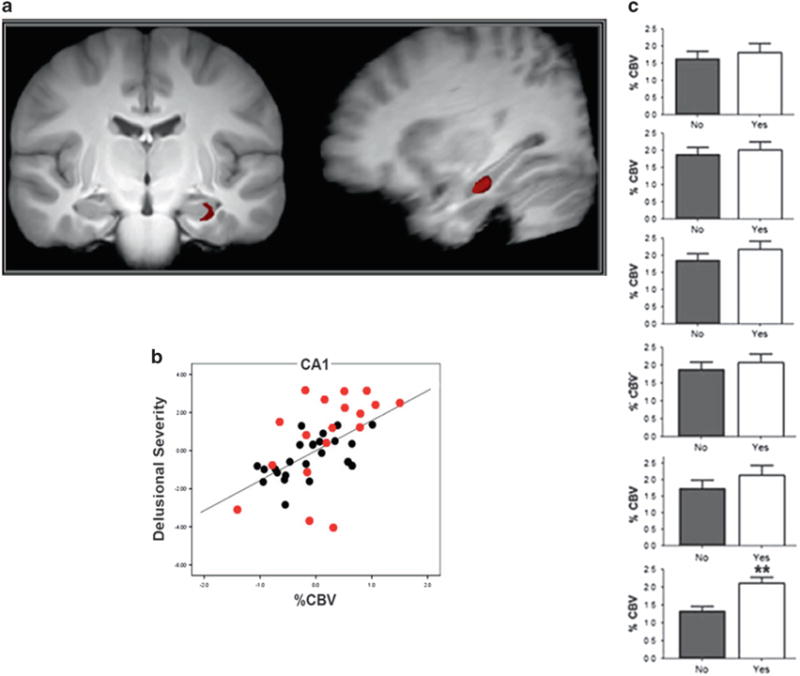
(**a**) Coronal and sagittal MR images showing specific left hippocampal CA1 subregion with elevated CBV in high risk patients as compared to healthy volunteers. (**b**) Scatterplot showing the relationship between psychotic symptoms (delusional severity, hallucinations not shown) and left hippocampal CA1 CBV in high risk (black) and schizophrenia (red) patients. (**c**) Bar graph of CBV values in left hippocampal CA1 subregion ranging from posterior (top) to anterior (bottom) showing significantly increased activity of latter in patients who progressed to psychotic disorders and those who did not.

Initial MRI assessments of high-risk subjects revealed elevated levels of hippocampal cerebral blood volume (CBV), a measure that is closely correlated with metabolism^37,38^ compared to demographically comparable healthy volunteers (Figure 1a). Moreover, higher CBV levels were positively correlated with the psychotic symptoms of subjects, specifically delusions and suspiciousness, in high-risk subjects and individuals with syndromal schizophrenia (Figure 1b).^19^

Subjects were reassessed 2 years of follow-up after their initial scan or sooner if they developed a syndromal psychotic disorder. Forty percent of the initial cohort (or 10 of 25 of the baseline sample) converted to a syndromal psychosis over the course of the follow-up period (mean time to conversion 339 days; median 323 days). (30% of the second cohort converted to a psychotic disorder in an average of 330 days). In these subjects, elevated baseline CBV in the CA1 region, and in particular left CA1 region, of the hippocampus, but no other hippocampal sub regions, was associated to conversion to psychosis (Figure 1c).^19,35^

We examined longitudinal changes in CBV between baseline and follow-up assessments, and found that: (1) CA1—CBV, which was higher in high-risk subjects compared to healthy volunteers, and in high-risk subjects who converted to psychosis compared to those that did not convert to psychosis, did not increase from baseline but remained elevated in relation to the healthy comparison group; (2) in subjects who developed psychotic disorders, CBV increased from baseline to conversion in the subiculum; no significant change in CBV occurred from time 1 to time 2 in other hippocampal sub-regions including entorhinal cortex, dentate gyrus, and CA3. Medication exposure had no effect on CBV values in this analysis.

## RESULTS

### Hippocampal structure

**Figure 2:**
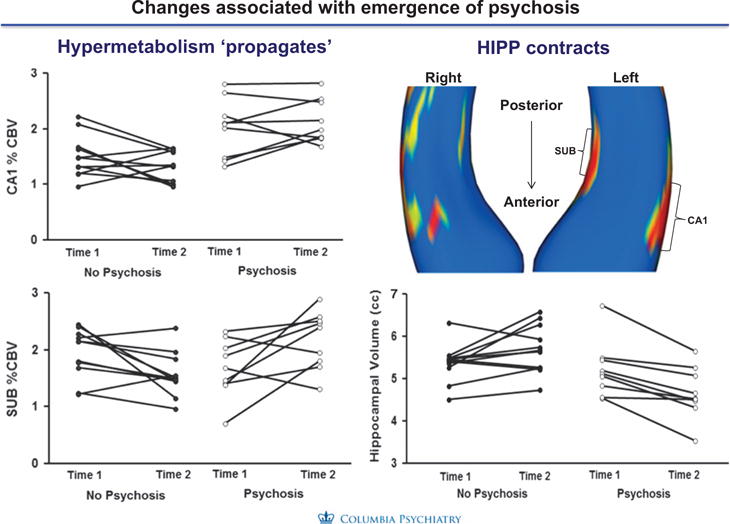
Relationship hippocampal CBV and atrophy. Hypermetabolism and atrophy of the hippocampus were strongly associated in anterior regions of the CA1 region but not in posterior or mid-regions.

MRI’s of the same subjects were used to map hippocampal structure and generate measures of hippocampal volume and hippocampal shape as previously described.^40–43^ There were no differences in hippocampal volume between clinical high risk and healthy subjects at their initial assessment. The hippocampal volume of high-risk subjects who subsequently developed psychotic disorders declined. Moreover, volume reduction among converters was localized to the CA1 and subiculum sub regions and were most prominent in the anterior left hippocampus (Figure 2).

Finally, we examined relationships between the increases in CA1 CBV and atrophy and found that the left anterior CA1 subfield hypermetabolism preceded and seemed to lead to morphologic shape and volume change reflected in the follow-up assessments as individuals progressed to psychosis (Supplementary Figure S3). These associations were not found in the posterior or mid body subfields. None of these changes were related to medication exposure.

### Examining glutamate as a pathogenic driver for metabolic and structural abnormalities

We hypothesized that the pathophysiology driving the anatomically concordant hypermetabolism and associated atrophy was caused by a dysregulation of glutamate neurotransmission and increased extracellular glutamate.^35^ This hypothesis derived from prior reports in the clinical and preclinical literature. Numerous case studies and anecdotal reports of the psychotomimetic effects of NMDA receptor blockers ketamine and phencyclidine showed that ketamine produces a behavioral syndrome that recapitulates the full spectrum of schizophrenia symptoms.^44–47^ These observations were consistent with the results of controlled studies in healthy humans in which administration of comparable ketamine doses produced similar behavioral effects and increases in limbic cortical metabolic activity.^44–47^ A seminal study by Moghaddam *et al.*^48^ found that systemic administration of the NMDA receptor antagonist MK801 led to an increase in extracellular glutamate in cerebral cortex, and that pharmacologically blocking this glutamate surge prevented the deleterious cognitive effects of the drug. Studies of NMDA receptor blockade on limbic metabolic activity produced homologous findings in rodents and humans, and provided a translational framework in which to examine the pathogenic mechanisms underlying the psychosis-related hippocampal CA1 hypermetabolism we had observed in patients.

### Clinical assessment of glutamate

Clinical studies of ^1H^MRS have found increased glutamate levels in prodromal and patients with schizophrenia in the hippocampus have found elevated levels in the hippocampus compared to healthy control subjects and associated with hypermetabolism in different brain regions of interest in patients with schizophrenia^28,29,48–50^ and including in some instances correlated with hippocampal atrophy.^29,48–50^.

Because of its importance as an excitatory neurotransmitter and potential for neurotoxicity, glutamate is tightly regulated by a group of enzymes distributed in astrocytes and neurons^51,52^—including glutamate dehydrogenase; glutaminase; glutamine synthetase; glutamic acid decarboxylase; GABA transaminase; and aspartate and alanine aminotransferases. It is therefore possible, that alterations in this group of enzymes might occur genetically, either directly, by genes encoding these enzymes;^53^ secondarily, by genetic links to the glutamatergic system^54–56^ or via environmental stressors and risk factors.^57^ Alternatively, alternations in this group of enzymes might occur via environmental stressors.

To examine this possibility, we used microarray to profile gene-expression levels of postmortem tissue collected from the CA1 subfield from the brains with schizophrenia and the age-matched controls, and the entorhinal cortex, a neighboring sub region of the hippocampal formation that was found to be differentially unaffected in schizophrenia.^58^ An ANOVA showed a significant ‘group × region’ interaction at a *P* < 0.005 for 19 transcripts (Supplementary Table S1). The most reliable change was a downregulation of glutamate dehydrogenase 1 (GLUD1; *P* = 0.00006). GLUD1 is expressed primarily in astrocytes where it is one of the main enzymes that degrades glutamate, and its deficiency might account for glutamate elevations.

### Ketamine recapitulates the psychosis-associated pattern of hippocampal dysfunction

**Figure 3:**
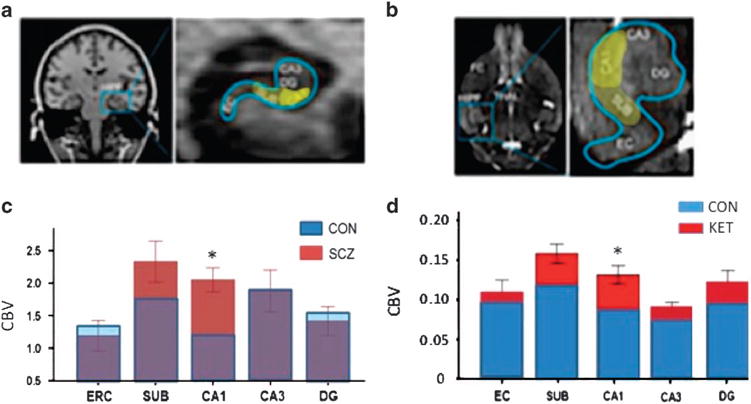
Regional patterns of increased metabolic activity in hippocampus of patients with schizophrenia (**a**, **c**) and induced by acute ketamine in mice (BD). (**a**) (Left) Coronal image of human brain showing location of hippocampus and (Right) enlarged image of hippocampus with yellow highlight of regions showing trending (subiculum) or significantly higher CBV values in schizophrenia patients relative to controls. (**b**) (Left) Horizontal image of mouse brain showing location of hippocampus and (Right) enlarged image of hippocampus with yellow highlight of regions showing trending (subiculum) or significantly higher CBV values in schizophrenia patients relative to controls. (**c, d**). Stacked bar graphs showing relative hyperactivity (increased CBV) in patients with schizophrenia relative to controls (**c**) following systemic ketamine (30 mg/kg) in mice (**d**). The pattern induced by ketamine with greatest deviation in CA1, then SUB, and non-significant in other regions. (Adapted from data in Schobel *et al.*^35^

To identify a possible role for fluxation and surges in extracellular glutamate driving CA1 hypermetabolism in psychosis, we examined changes in extracellular glutamate and CBV induced by acute systemic administration of the NMDA receptor antagonist ketamine (8–32 mg/kg) in parallel groups of C57/B6 mice.^35^ We measured glutamate using an amperometric glutamate sensor implanted into CA1 or other hippocampal sub regions of interest. CBV was measured using the same high-resolution contrast-based fMRI methods used in the clinical high-risk subjects. Ketamine induced increased hippocampal CBV in the same regional pattern of as seen in the human high-risk subjects (Figure 3). The sub region in which the ketamine challenge had the greatest effect on glutamate was CA1, a finding consistent with our previous study using the same CBV variant, suggesting that the CA1 region is specifically sensitive to alterations in glutamate.^59^

**Figure 4:**
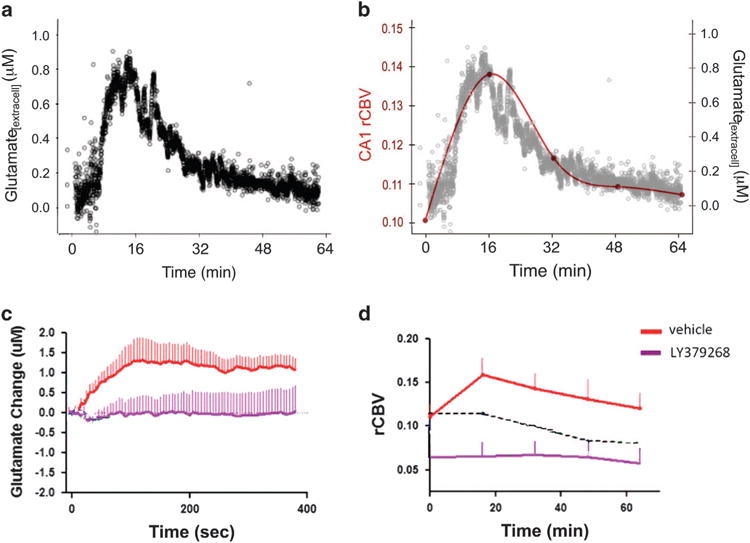
Parallel increases in extracellular glutamate and CBV following ketamine and blockade by the mGluR 2/3 agonist and glutamate release inhibitor LY379268. (**a**) Extracellular glutamate measured with amperometry. (**b**) Overlay of time courses of ketamine-induced increases of glutamate (black) and CBV (red line). (**c**) Relative to saline pretreatment (red line), LY379268 pretreatment (purple line) blocks ketamine-induced increases in extracellular glutamate. (**d**) Similar blockade effect of LY379268 pretreatment in ketamine-induced increase in CBV.

Next, to determine whether ketamine-induced increases in hippocampal metabolic activity were dependent on glutamate release, we tested the effects of a 5-day pre-treatment with LY 379286 (10 mg/kg per day), a metabotropic glutamate 2/3 receptor agonist that inhibits glutamate release. In the reference group pre-treated for 5 days with saline, acute ketamine evoked a robust increase in extracellular glutamate in CA1 exhibiting a similar time course as the observed increase in metabolic activity (Figures 4a and b). By contrast, pre-treatment for 5-days with the mGluR 2/3 agonist blocked both the ketamine-evoked glutamate efflux and increase in metabolic activity, reflected on MRI (Figures 4c and d). We also found that lamotrigine and gabapentin, treatments for seizure, mood and/or neuropathic pain disorders, both known to decrease indices of glutamate release, blocked ketamine-evoked increases in extracellular glutamate and metabolic activity (Supplementary Figure S4). Together, these experiments established that increases in extracellular glutamate can drive increases in metabolic activity within the hippocampal CA1 region and that can be mitigated by pharmacologic agents that inhibit glutamate.

### Episodic surges in extracellular glutamate as a driver of hippocampal hyperactivity and volume loss

**Figure 5:**
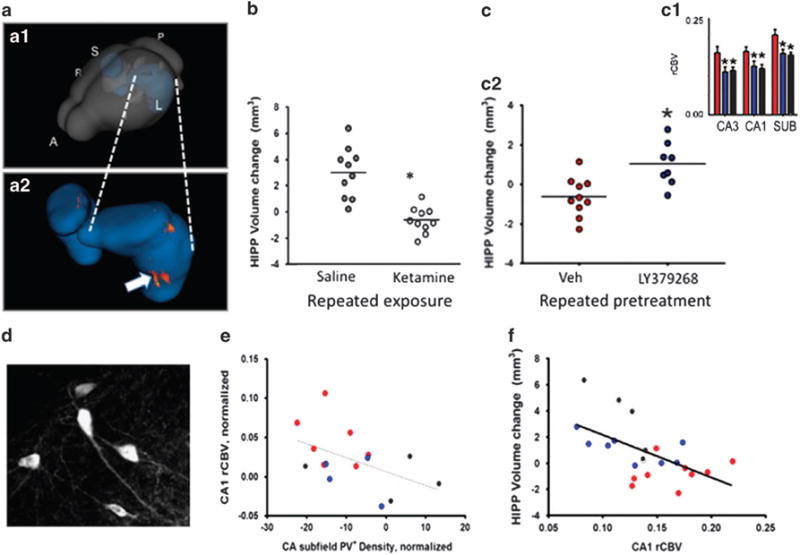
(**a**) Rendering of hippocampus within the mouse brain (A.1) and zoom-in of hippocampus (A.2) showing area of greatest morphological change produced by repeated ketamine treatment. (**b**) Repeated ketamine exposure (16 mg/kg, 3 × per week, 4 weeks)(open circles) blocks the normal growth of the hippocampus across the transition to adulthood in mice, leading to a reduction in volume relative to saline controls (gray circles). (**c**) Mice receiving repeated treatments with saline only (gray bars and circles), ketamine with saline pre-treatment (red) or ketamine with pre-treatment with the mGlurR 2/3 agonist LY347268 (blue). LY347268 co(pre)-treatment blocks the increase in basal CBV (**c1**) and relative loss of hippocampal volume (**c2**) produced by repeated ketamine exposure. (**d**). Parvalbumin expressing neurons in dorsal hippocampus of the mouse. (**e**) Correlation between PV+ neuron density and CA1 CBV; loss of PV+ interneurons is associated with increased CBV. (**f**). Hippocampal volume loss correlates with increases in CBV.

To test the possibility that the glutamate dysregulation-hypermetabolism dyad could contribute to the decreased hippocampal volume observed in schizophrenia patients, we modeled repeated “surges” of extracellular glutamate to determine the effect on hippocampal metabolic activity and volume. We administered saline or ketamine (8–32 mg/kg) to mice three times weekly for 1 month across the development period analogous to late adolescence-early adulthood in human. After a 2-day washout, we measured steady-state CBV and hippocampal volume (Figures 5a and b). Normally, hippocampal volume shows a developmental increase across the age range used in our study^60^, and this was observed in the saline-only treated control mice (Figure 5b). By contrast, mice exposed to repeated ketamine challenges showed a dose-dependent increase in “basal” metabolic activity (Figure 5C.1, (inset) and a decrease in hippocampal volume (Figure 5c), most notably the caudoventral CA1 and subiculum (Figure 5A.2,), regions homologous to the sub-region of greatest volume loss in our human study.^35^

### Linking glutamate dysregulation to cellular and structural pathology in hippocampal CA1

Finally, we tested whether pharmacologically inhibiting the acute increase in extracellular glutamate and metabolic activity stimulated by ketamine could mitigate the pathologic process that leads to a persistent increase in CA1 metabolic activity, down-regulation of PV+ interneurons, and loss of hippocampal volume. Co-treatment with the glutamate release inhibitor LY379268, administered prior to each of the 12 ketamine challenges (16 mg/kg) across one month, blocked CA1 CBV increases (Figure 5C.1, (inset) and mitigated hippocampal volume loss (Figure 5C.2,). With these findings demonstrating a link between glutamate dysregulation, CA1 hypermetabolism and hippocampal volume reduction, we next aimed to understand the pathological impact of the hyperglutamatergic-hypermetabolic events on hippocampal circuits. We started by examining parvalbumin-expressing (PV+) GABAergic interneurons (Figures 5d–f). These cells provide the major inhibitory input to glutamate secreting excitatory projection neurons of the hippocampus, tightly controlling their excitability and ultimately the output signal of the hippocampus.^1,61,62^ Extensive research in rodents provided both indirect and direct evidence that NMDA receptor antagonists can impair the function and reduce the viability of PV+ interneurons.^63–67^ These data and established theories of hippocampal interneuron function^67^ suggested the plausibility that a deficit in local inhibitory circuits could contribute to hippocampal glutamatergic dysregulation and hypermetabolism in psychosis.^1^ We found that repeated intermittent ketamine led to a modest but significant decrease in the density of detectable PV+ interneurons in the hippocampal CA fields.^35^ Notably, across groups exposed to repeated ketamine, ketamine with pretreatment with a glutamate release inhibitor, or no drug exposure, loss of PV+ interneurons correlated with the increase in basal CBV in CA1 after drug washout (Figure 5e); and, in turn, higher CA1 CBV predicted hippocampal volume loss (Figure 5f). We postulated that the repeated ketamine challenges produced a series of events in which a surge in extracellular glutamate drives metabolic demand. Over time, these events lead to a functional deficit and possible loss of viability of PV+ interneurons, which in turn may lead to a new higher set point for metabolic activity in hippocampal CA1, further toxicity to hippocampal cells and apparent shrinkage of anteromedial (or in rodent ventromedial) hippocampus. By limiting evoked glutamate efflux, the LY379268 treatment mitigated this pathogenic process.^68^

## DISCUSSION

The natural history of schizophrenia, characteristically evolves from a premorbid phase in which the clinical phenotype is not, or only partially, expressed through a series of stages culminating in the syndromal manifestation of symptoms meeting diagnostic criteria and constituting a first episode of a psychotic disorder. The subsequent course varies markedly based on illness severity, adequacy of treatment and environmental, including social, factors.

Numerous prior postmortem and neuroimaging studies have demonstrated early pathological involvement of the hippocampal formation in schizophrenia (as reviewed above). These findings implicate the fundamental relationship of the hippocampus to the onset and early course of schizophrenia as reflected by measures of its metabolism and structure. Our longitudinal study of clinical high-risk patients revealed a specific spatiotemporal pattern of hippocampal dysfunction that progresses in the transition from attenuated psychotic symptoms to syndromal psychosis. During pre-syndromal stages or attenuated psychotic symptoms, increased glutamate levels and hypermetabolism of hippocampal neurons selectively occurs in the CA1 sub-region. Those patients who progress go through the prodromal stage to syndromal psychosis, this pathologic process spreads from CA1 to the subiculum (and likely beyond the hippocampus) and leads to hippocampal volume reduction in a precise spatially concordant manner.

We tested this pathophysiological hypothesis in a rodent model with three experiments. First, we found that ketamine-evoked increases in extracellular glutamate in mice mirrored the evoked fMRI pattern, with maximal changes found in the CA1 and subiculum hippocampal sub regions. Second, we demonstrated that the ketamine induced effects described were associated with (and presumably caused) atrophy in a spatial-temporally concordant manner. Third, we established a mechanistic link by pretreating with an agent that inhibited or blocked ketamine-induced extracellular glutamate efflux, hypermetabolism and spatiotemporally concordant atrophy in the hippocampus. Finally, we extended this acute experiment longitudinally over 1 month and found that by inhibiting extracellular glutamate efflux with therapeutic agents prior to intermittent ketamine administration, sustained basal hypermetabolism and hippocampal atrophy were reduced or prevented.

The mGluR 2/3 agonists were an optimal choice to provide this mechanistic link because of their selectivity in limiting glutamate efflux, and their ability to block the behavioral and cognitive abnormalities produced by psychotomimetic drugs.^69–73^ In our experiments, LY379268 potently blocked both the evoked imaging and neurochemical responses, supporting the hypothesis that increases in extracellular glutamate are necessary to evoke hippocampal hypermetabolism and hippocampal atrophy.

Numerous studies by other investigators and from other laboratories support the hypothesis that glutamate neurotransmission plays a critical role in mediating the cognitive and behavioral disturbances of psychosis.^74,75^ In the context of a pharmacologic model of NMDA receptor hypofunction, NMDA receptor antagonist administration has been shown to increase glutamate efflux in part by decreased excitatory drive of GABAergic inhibitory interneurons, thereby increasing the activity of glutamatergic neurons^63,76,77^ and increasing metabolic demand and blood flow.^78,79^ This sequence of effects is likely to occur in the hippocampus. Differential regional vulnerability to the effects of NMDA receptor blockade within the hippocampal circuit may be mediated at a molecular level by increased density of NMDA and AMPA receptors in CA1 relative to the CA3 subfield, dentate gyrus and entorhinal cortex;^79^ and AMPA receptors may play an important role in the consequent synaptic and hemodynamic state.^73^ Further research is needed to relate these findings to neuropathological findings in the hippocampus in schizophrenia, which have shown abnormalities in AMPA/kainate and NMDA receptors,^80–83^ GABA receptors and interneurons.^3^ However, these molecular findings in neuropathological samples, observed mainly at the level of gene expression in regions that provide excitatory input to CA1,^84^ have been postulated to have a significant impact on activity within CA1, a hypothesis consistent with a number of findings.^2,19,85^ These findings, taken together, support the hypothesis that glutamate elevation drives hypermetabolism and atrophy in the CA1 and subiculum sub-regions of the hippocampus.^86,87^

In addition to clarifying mechanisms of disease onset, the aforementioned studies indicate that measures of increases of extracellular glutamate in CA1, may serve as a state-specific biomarker of prodromal psychotic disorders. As with other progressive disorders of the brain, such as Alzheimer’s disease, for example, early detection and treatment during prodromal stages, when the disease is restricted to relatively confined areas of the brain, has emerged as an important therapeutic strategy in alleviating symptoms and disease modification. By showing that hypermetabolism occurs before atrophy, our results reinforce this concept, because reversing functional defects are likely easier before the development of structural brain pathology. In addition, these results suggest that reducing extracellular glutamate is a valid target for preventing or ameliorating the onset of illness and limiting hippocampal atrophy, one of the first regions of the brain to show volumetric loss in schizophrenia.^21^ Notably, glutamate-reducing agents include approved drugs such as lamotrigine or gabapentin, as well as the experimental compound pomaglumetad (LY404309). It is possible, therefore, to design a study in subjects at high risk for psychotic disorders to test whether these drugs normalize hippocampal hypermetabolism and prevent progression to psychosis, and hippocampal atrophy.

Although the source of glutamate dysregulation awaits further exploration, the imaging studies summarized earlier suggest that glutamate elevation itself is a valid drug target and that glutamate-reducing agents might be effective for therapeutic intervention. An important implication of the imaging studies is that these agents should be given during prodromal stages of disease, before its imaging correlate of atrophy and the loss of interneurons occurs. Indeed, recent failures of clinical trials using the glutamate-reducing agent pomaglumetad (LY2140023)^88^ might be due to the fact that they were tested in patients who were already in advanced stages of the disease as has been suggested as the reasons for the failure of other treatments such as the anti-amyloid vaccine therapies for Alzheimer’s disease.^89^

## Supplementary Material

suppfig1

suppfig2

suppfig3

suppfig4

supptable1

## References

[1] Lewis DA, Hashimoto T, Volk DW (2005). Cortical inhibitory neurons and schizophrenia. Nat Rev Neurosci.

[2] Benes FM (1999). Evidence for altered trisynaptic circuitry in schizophrenic hippocampus. Biol Psychiatry.

[3] Heckers S, Konradi C (2015). GABAergic mechanisms of hippocampal hyperactivity in schizophrenia. Schizophr Res.

[4] Bogerts B, Ashtari M, Degreef G, Alvir JM, Bilder RM, Lieberman JA (1990). Reduced temporal limbic structure volumes on magnetic resonance images in first episode schizophrenia. Psychiatry Res.

[5] Kendler KS (2013). What psychiatric genetics has taught us about the nature of psychiatric illness and what is left to learn. Mol Psychiatry.

[6] Harrison PJ (2015). Recent genetic findings in schizophrenia and their therapeutic relevance. J Psychopharmacol.

[7] Green IW, Glausier JR (2016). Different paths to core pathology: the equifinal model of the schizophrenia syndrome. Schizophr Bull.

[8] Lieberman J, Jody D, Geisler S, Alvir J, Loebel A, Szymanski S (1993). Time course and biological correlates of treatment response in first episode schizophrenia. Arch General Psychiatry.

[9] Wyatt RJ (1991). Early intervention with neuroleptics may decrease the long-term morbidity of schizophrenia. Schizophr Res.

[10] Wyatt RJ (1991). Neuroleptics and the natural course of schizophrenia. Schizophr Bull.

[11] Loebel AD, Lieberman JA, Alvir JM, Mayerhoff DI, Geisler SH, Szymanski SR (1992). Duration of psychosis and outcome in first-episode schizophrenia. Am J Psychiatry.

[12] Perkins DO, Gu H, Boteva K, Lieberman JA (2005). Relationship between duration of untreated psychosis and outcome in first-episode schizophrenia: a critical review and meta-analysis. Am J Psychiatry.

[13] Lieberman JA, Perkins D, Belger A, Chakos M, Jarskog F, Boteva K (2001). The early stages of schizophrenia: speculations on pathogenesis, pathophysiology, and therapeutic approaches. Biol Psychiatry.

[14] Dixon LB, Goldman HH, Bennett ME, Wang Y, McNamara KA, Mendon SJ (2015). Implementing coordinated specialty care for early psychosis: the RAISE connection program. Psychiatr Serv.

[15] Addington J, Cadenhead KS, Cannon TD, Cornblatt B, McGlashan TH, Perkins DO (2007). North American Prodrome Longitudinal Study: a collaborative multisite approach to prodromal schizophrenia research. Schizophrenia Bull.

[16] Lieberman J, Corcoran CM (2007). The impossible dream: can psychiatry prevent psychosis?. Early Interv Psychiatry.

[17] Fusar-Poli P, Borgwardt S, Bechdolf A, Addington J, Richer-Rossler A, Schultze-Lutter F (2013). The psychosis high-risk state: a comprehensive state-of-the-art review. JAMA Psychiatry.

[18] Kuhn S, Musso F, Mobascher A, Warbrick T, Winterer G, Gallinat J (2012). Hippocampal subfields predict positive symptoms in schizophrenia: first evidence from brain morphometry. Translational Psychiatry.

[19] Schobel SA, Lewandowski NM, Corcoran CM, Moore H, Brown T, Malaspina D (2009). Differential targeting of the CA1 subfield of the hippocampal formation by schizophrenia and related psychotic disorders. Arch Gen Psychiatry.

[20] Csernansky JG, Joshi S, Wang L, Haller JW, Gado M, Miller JP (1998). Hippocampal morphometry in schizophrenia by high dimensional brain mapping. Proc Natl Acad Sci USA.

[21] Steen RG, Mull C, McClure R, Hamer RM, Lieberman JA (2006). Brain volume in first-episode schizophrenia: systematic review and meta-analysis of magnetic resonance imaging studies. Br J Psychiatry.

[22] Molina V, Reig S, Pascau J, Sanz J, Sarramea F, Gispert JD (2003). Anatomical and functional cerebral variables associated with basal symptoms but not risperidone response in minimally treated schizophrenia. Psychiatry Res.

[23] Medoff DR, Holcomb HH, Lahti AC, Tamminga CA (2001). Probing the human hippocampus using rCBF: contrasts in schizophrenia. Hippocampus.

[24] Heckers S, Rauch SL, Goff D, Savage CR, Schacter DL, Fischman AJ (1998). Impaired recruitment of the hippocampus during conscious recollection in schizophrenia. Nat Neurosci.

[25] Kawasaki Y, Suzuki M, Maeda Y, Urata K, Yamaguchi N, Matsuda H (1992). Regional cerebral blood flow in patients with schizophrenia. A preliminary report Eur Arch Psychiatry Clin Neurosci.

[26] Malaspina D, Harkavy-Friedman J, Corcoran C, Mujica-Parodi L, Printz D, Gorman JM (2004). Resting neural activity distinguishes subgroups of schizophrenia patients. Biol Psychiatry.

[27] Narr KL, Thompson PM, Szeszko P, Robinson D, Jang S, Woods RP (2004). Regional specificity of hippocampal volume reductions in first-episode schizophrenia. Neuroimage.

[28] Ho NF, Iglesias JE, Sum MY, Kuswanto CN, Sitoh YY, De Souza J (2017). Progression from selective to general involvement of hippocampal subfields in schizophrenia. Mol Psychiatry.

[29] Kraguljac NV, Frölich MA, Tran S, White DM, Nichols N, Barton-McArdle A (2017). Ketamine modulates hippocampal neurochemistry and functional connectivity: a combined magnetic resonance spectroscopy and resting-state fMRI study in healthy volunteers. Mol Psychiatry.

[30] Shi X, Ibrahim JG, Lieberman J, Styner M, Zhu H (2011). Two-stage empirical likelihood for longitudinal neuroimaging data. Ann Appl Stat.

[31] Lieberman JA (1999). Is schizophrenia a neurodegenerative disorder? A clinical and neurobiological perspective. Biol Psychiatry.

[32] DeLisi LE (1999). Regional brain volume change over the life-time course of schizophrenia. J Psychiatr Res.

[33] Andreasen NC, Nopoulos P, Magnotta V, Pierson R, Ziebell S, Ho BC (2011). Progressive brain change in schizophrenia: a prospective longitudinal study of first-episode schizophrenia. Biol Psychiatry.

[34] Miller TJ, McGlashan TH, Rosen JL, Cadenhead K, Ventura J, McFarlane W (2003). Prodromal assessment with the structured interview for prodromal syndromes and the scale of prodromal symptoms: predictive validity, interrater reliability, and training to reliability. Schizophr Bull.

[35] Schobel SA, Chaudhury NH, Khan UA, Paniagua B, Styner MA, Asllani I (2013). Imaging patients with psychosis and a mouse model establishes a spreading pattern of hippocampal dysfunction and implicates glutamate as a driver. Neuron.

[36] Small SA (2014). Isolating pathogenic mechanisms embedded within the hippocampal circuit through regional vulnerability. Neuron.

[37] Raichle ME (1983). Positron emission tomography. Annu Rev Neurosci.

[38] Gonzalez RG, Fischman AJ, Guimaraes AR, Carr CA, Stern CE, Halpern EF (1995). Functional MR in the evaluation of dementia: correlation of abnormal dynamic cerebral blood volume measurements with changes in cerebral metabolism on positron emission tomography with fludeoxyglucose F 18. AJNR Am J Neuroradiol.

[39] Cannon TD, Cadenhead K, Cornblatt B, Woods SW, Addington J, Walker E (2008). Prediction of psychosis in youth at high clinical risk: a multisite longitudinal study in North America. Arch Gen Psychiatry.

[40] Chakos MH, Schobel SA, Gu H, Gerig G, Bradford D, Charles C (2005). Duration of illness and treatment effects on hippocampal volume in male patients with schizophrenia. Br J Psychiatry.

[41] Schobel SA, Kelly MA, Corcoran CM, Van Heertum K, Seckinger R, Goetz R (2009). Anterior hippocampal and orbitofrontal cortical structural brain abnormalities in association with cognitive deficits in schizophrenia. Schizophr Res.

[42] Pruessner JC, Li LM, Serles W, Pruessner M, Collins DL, Kabani N (2000). Volumetry of hippocampus and amygdala with high-resolution MRI and three-dimensional analysis software: minimizing the discrepancies between laboratories. Cereb Cortex.

[43] Styner M, Gerig G, Lieberman J, Jones D, Weinberger D (2003). Statistical shape analysis of neuroanatomical structures based on medial models. Med Image Anal.

[44] Krystal JH, Karper LP, Seibyl JP, Freeman GK, Delaney R, Bremner JD (1994). Sub-anesthetic effects of the noncompetitive NMDA antagonist, ketamine, in humans. Psychotomimetic, perceptual, cognitive, and neuroendocrine responses. Arch Gen Psychiatry.

[45] Lahti AC, Holcomb HH, Medoff DR, Tamminga CA (1995). Ketamine activates psychosis and alters limbic blood flow in schizophrenia. Neuroreport.

[46] Holcomb HH, Lahti AC, Medoff DR, Weiler M, Tamminga CA (2001). Sequential regional cerebral blood flow brain scans using PET with H2(15)O demonstrate ketamine actions in CNS dynamically. Neuropsychopharmacology.

[47] Vollenweider FX, Leenders KL, Scharfetter C, Antonini A, Maguire P, Missimer J (1997). Metabolic hyperfrontality and psychopathology in the ketamine model of psychosis using positron emission tomography (PET) and [18F] fluorodeoxyglucose (FDG). Eur Neuropsychopharmacol.

[48] Moghaddam B, Javitt D (2012). From revolution to evolution: the glutamate hypothesis of schizophrenia and its implication for treatment. Neuropsychopharmacology.

[49] Kraguljac NV, White DM, Reid MA, Lahti AC (2013). Increased hippocampal glutamate and volumetric deficits in unmedicated patients with schizophrenia. JAMA Psychiatry.

[50] Kegeles LS, Mao X, Stanford AD, Girgis R, Ojeil N, Xu X (2012). Elevated prefrontal cortex γ-aminobutyric acid and glutamate-glutamine levels in schizophrenia measured in vivo with proton magnetic resonance spectroscopy. Arch Gen Psychiatry.

[51] Maciejewski PK, Rothman DL (2008). Proposed cycles for functional glutamate trafficking in synaptic neurotransmission. Neurochem Int.

[52] Erecinska M, Silver IA (1990). Metabolism and role of glutamate in mammalian brain. Prog Neurobiol.

[53] Jia P, Wang L, Meltzer HY, Zhao Z (2010). Common variants conferring risk of schizophrenia: a pathway analysis of GWAS data. Schizophr Res.

[54] Winchester CL, Pratt JA, Morris BJ (2014). Risk genes for schizophrenia: translational opportunities for drug discovery. Pharmacol Ther.

[55] Wilson GM, Flibotte S, Chopra V, Melnyk BL, Honer WG, Holt RA (2006). DNA copy-number analysis in bipolar disorder and schizophrenia reveals aberrations in genes involved in glutamate signaling. Hum Mol Genet.

[56] Walsh T, McClellan JM, McCarthy SE, Addington AM, Pierce SB, Cooper GM (2008). Rare structural variants disrupt multiple genes in neurodevelopmental pathways in schizophrenia. Science.

[57] Matrisciano F, Tueting P, Maccari S, Nicoletti F, Guidotti A (2012). Pharmacological activation of group-II metabotropic glutamate receptors corrects a schizophrenia-like phenotype induced by prenatal stress in mice. Neuropsychopharmacology.

[58] Lewandowski N (2008). Imaging-guided microarray identifies molecular markers in schizophrenia and Parkinson’s Disease. PhD thesis.

[59] Gaisler-Salomon I, Miller GM, Chuhma N, Lee S, Zhang H, Ghoddoussi F (2009). Glutaminase-deficient mice display hippocampal hypoactivity, insensitivity to pro-psychotic drugs and potentiated latent inhibition: relevance to schizophrenia. Neuropsychopharmacology.

[60] Hammelrath L, Škokić S, Khmelinskii A, Hess A, van der Knaap N, Staring M (2016). Morphological maturation of the mouse brain: an in vivo MRI and histology investigation. NeuroImage.

[61] Lee SH, Marchionni I, Bezaire M, Varga C, Danielson N, Lovett-Barron M (2014). Parvalbumin-positive basket cells differentiate among hippocampal pyramidal cells. Neuron.

[62] Buzsaki G, Wang XJ (2012). Mechanisms of gamma oscillations. Annu Rev Neurosci.

[63] Behrens MM, Ali SS, Dao DN, Lucero J, Shekhtman G, Quick KL (2007). Ketamine-induced loss of phenotype of fast-spiking interneurons is mediated by NADPH-oxidase. Science.

[64] Keilhoff G, Becker A, Grecksch G, Wolf G, Bernstein HG (2004). Repeated application of ketamine to rats induces changes in the hippocampal expression of parvalbumin, neuronal nitric oxide synthase and cFOS similar to those found in human schizophrenia. Neuroscience.

[65] Vutskits L, Gascon E, Potter G, Tassonyi E, Kiss JZ (2007). Low concentrations of ketamine initiate dendritic atrophy of differentiated GABAergic neurons in culture. Toxicology.

[66] Kinney JW, Davis CN, Tabarean I, Conti B, Bartfai T, Behrens MM (2006). A specific role for NR2A-containing NMDA receptors in the maintenance of parvalbumin and GAD67 immunoreactivity in cultured interneurons. J Neurosci.

[67] de Lima AD, Opitz T, Voigt T (2004). Irreversible loss of a subpopulation of cortical interneurons in the absence of glutamatergic network activity. Eur J Neurosci.

[68] Lisman JE, Coyle JT, Green RW, Javitt DC, Benes FM, Heckers S (2008). Circuit-based framework for understanding neurotransmitter and risk gene interactions in schizophrenia. Trends Neurosci.

[69] Moghaddam B, Adams BW (1998). Reversal of phencyclidine effects by a group II metabotropic glutamate receptor agonist in rats. Science.

[70] Moghaddam B (2003). Bringing order to the glutamate chaos in schizophrenia. Neuron.

[71] Krystal JH, Abi-Saab W, Perry E, D’Souza DC, Liu N, Gueorguieva R (2005). Preliminary evidence of attenuation of the disruptive effects of the NMDA glutamate receptor antagonist, ketamine, on working memory by pretreatment with the group II metabotropic glutamate receptor agonist, LY354740, in healthy human subjects. Psychopharmacology.

[72] Cartmell J, Monn JA, Schoepp DD (1999). The metabotropic glutamate 2/3 receptor agonists LY354740 and LY379268 selectively attenuate phencyclidine versus d-amphetamine motor behaviors in rats. J Pharmacol Exp Ther.

[73] Imre G, Salomons A, Jongsma M, Fokkema DS, Den Boer JA, Ter Horst GJ (2006). Effects of the mGluR2/3 agonist LY379268 on ketamine-evoked behaviours and neurochemical changes in the dentate gyrus of the rat. Pharmacol Biochem Behav.

[74] Moghaddam B, Adams B, Verma A, Daly D (1997). Activation of glutamatergic neurotransmission by ketamine: a novel step in the pathway from NMDA receptor blockade to dopaminergic and cognitive disruptions associated with the prefrontal cortex. J Neurosci.

[75] Patil ST, Zhang L, Martenyi F, Lowe SL, Jackson KA, Andreev BV (2007). Activation of mGlu2/3 receptors as a new approach to treat schizophrenia: a randomized Phase 2 clinical trial. Nat Med.

[76] Greene R (2001). Circuit analysis of NMDAR hypofunction in the hippocampus, in vitro, and psychosis of schizophrenia. Hippocampus.

[77] Homayoun H, Moghaddam B (2007). NMDA receptor hypofunction produces opposite effects on prefrontal cortex interneurons and pyramidal neurons. J Neurosci.

[78] Rothman DL, Behar KL, Hyder F, Shulman RG (2003). In vivo NMR studies of the glutamate neurotransmitter flux and neuroenergetics: implications for brain function. Annu Rev Physiol.

[79] Pellerin L, Magistretti PJ (2004). Neuroenergetics: calling upon astrocytes to satisfy hungry neurons. Neuroscientist.

[80] Coultrap SJ, Nixon KM, Alvestad RM, Valenzuela CF, Browning MD (2005). Differential expression of NMDA receptor subunits and splice variants among the CA1, CA3 and dentate gyrus of the adult rat. Brain Res Mol Brain Res.

[81] Gao XM, Sakai K, Roberts RC, Conley RR, Dean B, Tamminga CA (2000). Ionotropic glutamate receptors and expression of N-methyl-D-aspartate receptor subunits in subregions of human hippocampus: effects of schizophrenia. Am J Psychiatry.

[82] Harrison PJ, McLaughlin D, Kerwin RW (1991). Decreased hippocampal expression of a glutamate receptor gene in schizophrenia. Lancet.

[83] Law AJ, Deakin JF (2001). Asymmetrical reductions of hippocampal NMDAR1 glutamate receptor mRNA in the psychoses. Neuroreport.

[84] Harrison PJ, Law AJ, Eastwood SL (2003). Glutamate receptors and transporters in the hippocampus in schizophrenia. Ann N Y Acad Sci.

[85] Harrison PJ (2004). The hippocampus in schizophrenia: a review of the neuropathological evidence and its pathophysiological implications. Psychopharmacology.

[86] Stone JM, Dietrich C, Edden R, Mehta MA, De Simoni S, Reed LJ (2012). Ketamine effects on brain GABA and glutamate levels with 1H-MRS: relationship to ketamine-induced psychopathology. Mol Psychiatry.

[87] de la Fuente-Sandoval C, Leon-Ortiz P, Favila R, Stephano S, Mamo D, Ramirez-Bermudez J (2011). Higher levels of glutamate in the associative-striatum of subjects with prodromal symptoms of schizophrenia and patients with first-episode psychosis. Neuropsychopharmacology.

[88] Adams DH, Zhang L, Millen BA, Kinon BJ, Gomez JC (2014). Pomaglumetad Methionil (LY2140023 Monohydrate) and Aripiprazole in Patients with Schizophrenia: A Phase 3, Multicenter, Double-Blind Comparison. Schizophr Res Treatment.

[89] Wisniewski T, Goni F (2015). Immunotherapeutic approaches for Alzheimer’s disease. Neuron.

