# Supplementary figures and images for: Hippocampal dysfunction in the pathophysiology of schizophrenia: a selective review and hypothesis for early detection and intervention
Source: Mol Psychiatry. Author manuscript; Available in PMC 2019 Aug 1. (PMC6037569; doi:10.1038/mp.2017.249)

## Slide 1
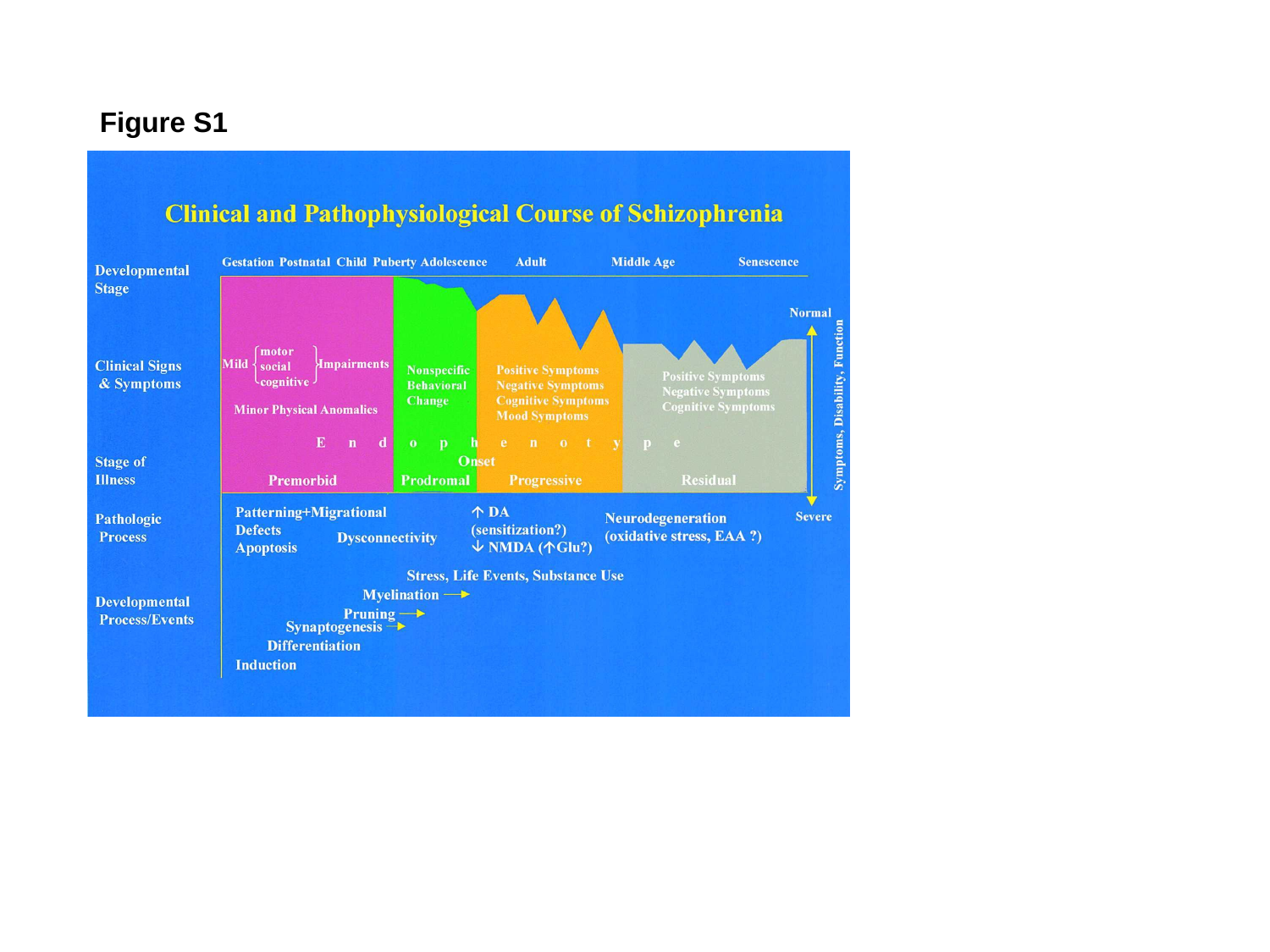

# Figure S1

Supplement: suppfig1 [file NIHMS956004-supplement-suppfig1.ppt]

## Slide 1
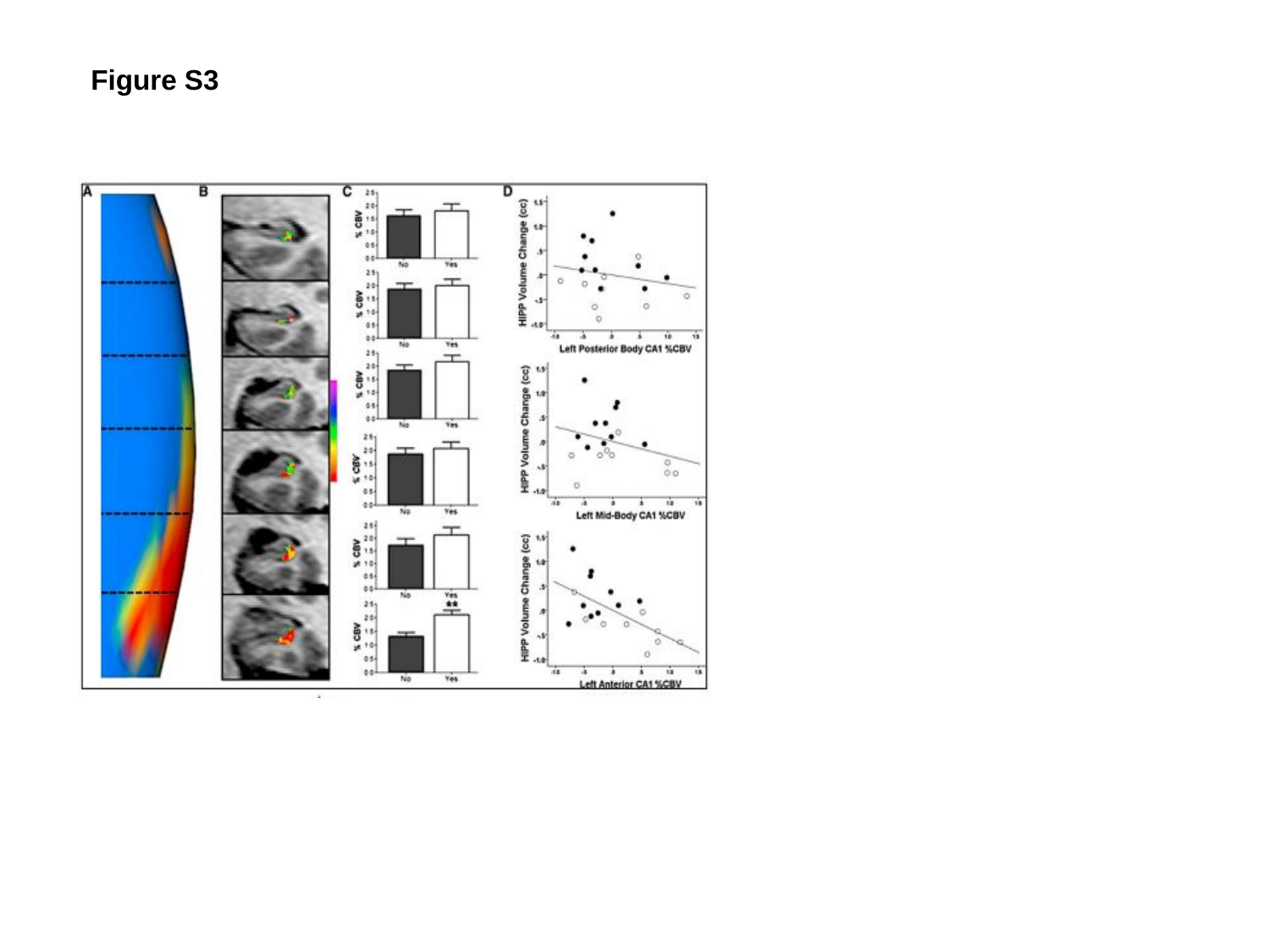

# Figure S3

Supplement: suppfig3 [file NIHMS956004-supplement-suppfig3.ppt]
