# Supplementary material for: Hippocampal dysfunction in the pathophysiology of schizophrenia: a selective review and hypothesis for early detection and intervention
Source: Mol Psychiatry. Author manuscript; Available in PMC 2019 Aug 1. (PMC6037569; doi:10.1038/mp.2017.249)
Supplement: suppfig2 [file NIHMS956004-supplement-suppfig2.ppt]

## Slide 1
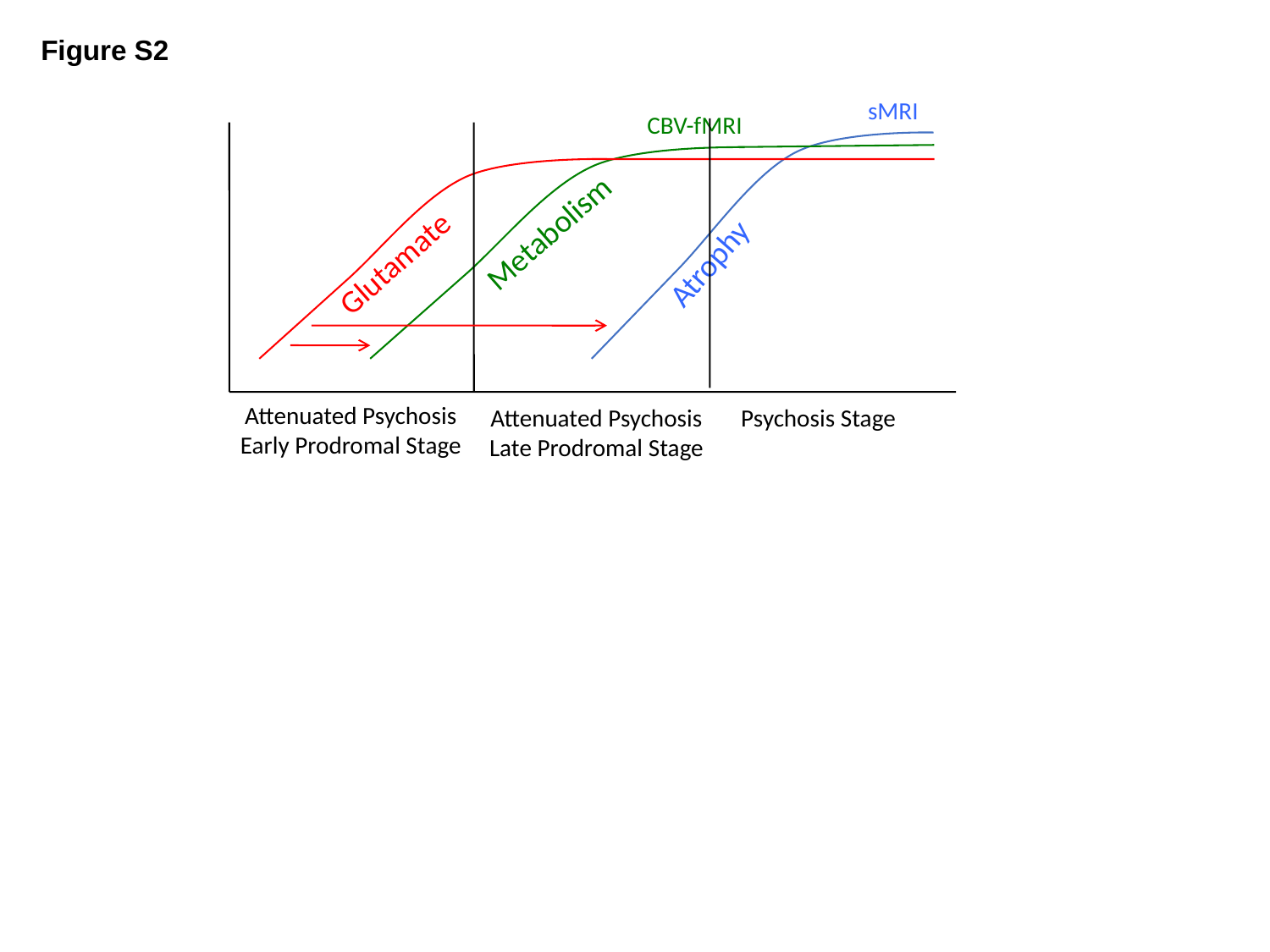

Figure S2
sMRI
CBV-fMRI
Metabolism
Atrophy
Glutamate
Attenuated Psychosis
Early Prodromal Stage
Attenuated Psychosis
Late Prodromal Stage
Psychosis Stage
