# Supplementary material for: Hippocampal dysfunction in the pathophysiology of schizophrenia: a selective review and hypothesis for early detection and intervention
Source: Mol Psychiatry. Author manuscript; Available in PMC 2019 Aug 1. (PMC6037569; doi:10.1038/mp.2017.249)
Supplement: suppfig4 [file NIHMS956004-supplement-suppfig4.ppt]

## Slide 1
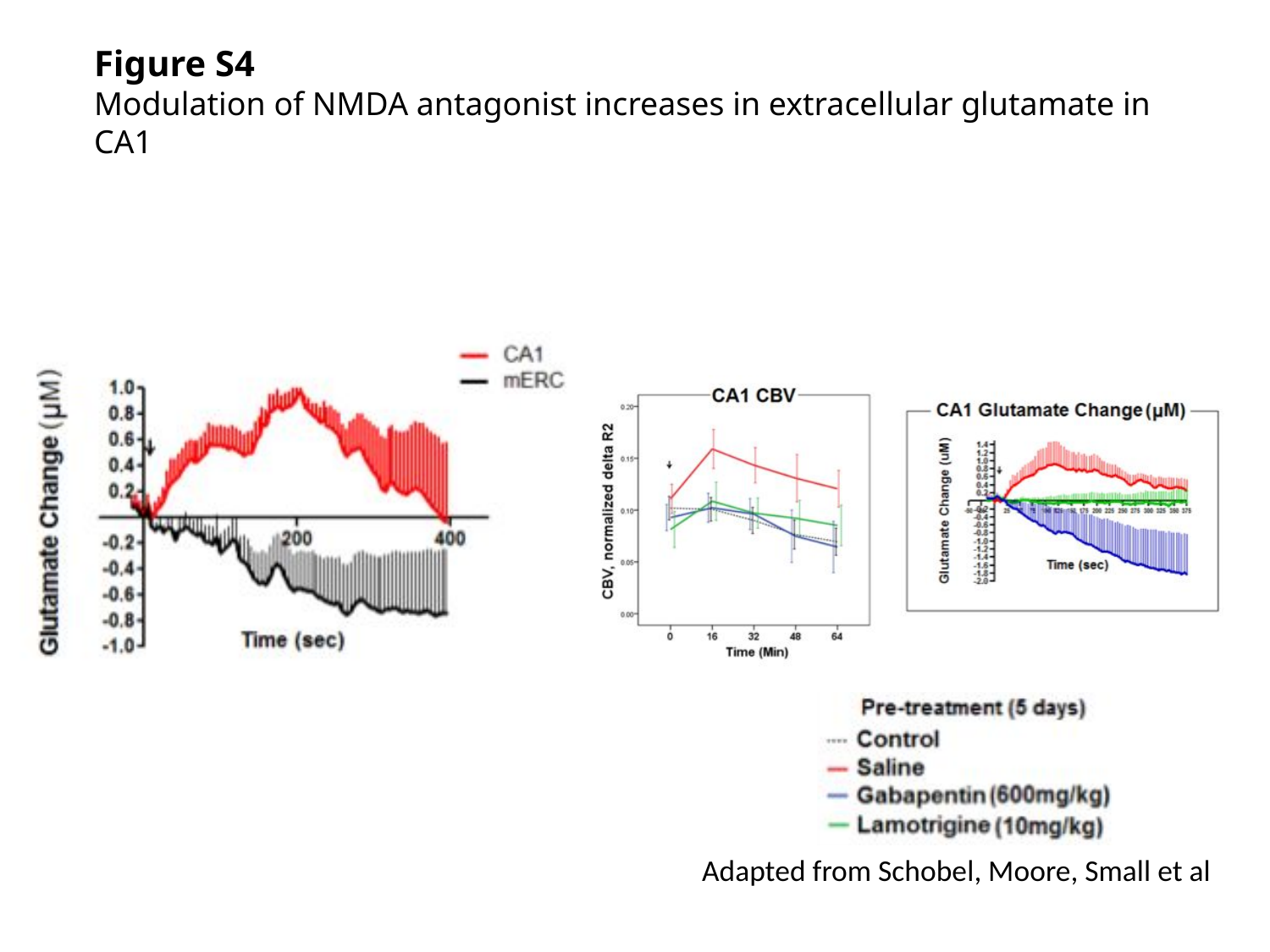

Figure S4
Modulation of NMDA antagonist increases in extracellular glutamate in CA1
Adapted from Schobel, Moore, Small et al
