# Supplementary material for: Hippocampal dysfunction in the pathophysiology of schizophrenia: a selective review and hypothesis for early detection and intervention
Source: Mol Psychiatry. Author manuscript; Available in PMC 2019 Aug 1. (PMC6037569; doi:10.1038/mp.2017.249)
Supplement: supptable1 [file NIHMS956004-supplement-supptable1.docx]

| **Gene Name** | **GenBank Accession #** | **p value^¥^** | **Cal* schiz** | **Cal* control** |
| --- | --- | --- | --- | --- |
| glutamate dehydrogenase 1 | NM_005271.1 | 0.00006 | 642 | 1062 |
| FU20502 | NM_017845.1 | 0.00058 | 329 | 598 |
| Tachykinin 2 precursor, isoform beta | NM_003182.1 | 0.00069 | 832 | 423 |
| alpha enolase like 1 | U88968.l | 0.00083 | 1098 | 521 |
| immunoglobulin kappa orphan gene | X51887 | 0.00085 | 1138 | 728 |
| X-prolyl aminopeptidase-like | NM_006523.1 | 0.00094 | 698 | 409 |
| tweety (Drosophila) homolog 1 | NM_020659.1 | 0.00094 | 613 | 392 |
| CBl cannabinoid receptor | U73304 | 0.0012 | 439 | 279 |
| sialyltransferase 4A | NM_003033.1 | 0.0013 | 1214 | 763 |
| zinc finger protein 232 | NM_014519.1 | 0.0019 | 1189 | 661 |
| microtubule-associated protein like echinoderm | NM_012155.l | 0.0019 | 2791 | 1454 |
| Eukaryotic translation initiation factor   A | NM_004681.1 | 0.0024 | 1017 | 686 |
| ephrin-A4 | NM_005227.1 | 0.0030 | 3541 | 1760 |
| KIAA1547 protein | Al567426 | 0.0030 | 3186 | 1722 |
| progesterone binding protein | AL547946 | 0.0034 | 671 | 366 |
| antiquitin 1 | BC002515.1 | 0.0035 | 1138 | 728 |
| cathepsin Z | AA418800 | 0.0036 | 2152 | 986 |
| DKFZp434C198 | AL136896.1 | 0.0039 | 1042 | 656 |
| tetraspan 3 | BC000704.l | 0.0045 | 4535 | 2424 |

**Table S1**
